# Spatiotemporal expression of histone acetyltransferases, p300 and CBP, in developing embryonic hearts

**DOI:** 10.1186/1423-0127-16-24

**Published:** 2009-02-23

**Authors:** Guozhen Chen, Jing Zhu, Tiewei Lv, Gang Wu, Huichao Sun, Xupei Huang, Jie Tian

**Affiliations:** 1 Department of Cardiology, The Children's Hospital of Chongqing Medical University, Chongqing, PR China; 2 Department of Basic Science, Charlie E. Schmidt College of Biomedical Science, Florida Atlantic University, Boca Raton, FL, USA

## Abstract

Histone acetyltransferases (HATs), p300 and cAMP response element binding protein (CREB)-binding protein (CBP) are two structurally related transcriptional co-activators that activate expression of many eukaryotic genes involved in cellular growth and signaling, muscle differentiation and embryogenesis. However, whether these proteins play important and different roles in mouse cardiogenesis is not clear. Here, we investigate the protein distributions and mRNA expression of the two HATs in embryonic and adult mouse heart during normal heart development by using immunohistochemical and RT-PCR techniques. The data from immunohistochemical experiments revealed that p300 was extensively present in nearly every region of the hearts from embryonic stages to the adulthood. However, no CBP expression was detected in embryonic hearts at day E7.5. CBP expression appeared at the later stages, and the distribution of CBP was less than that of p300. In the developmental hearts after E10.5, both for p300 and CBP, the mRNA expression levels reached a peak on day E10.5, and then were gradually decreased afterwards. These results reveal that both p300 and CBP are related to embryonic heart development. The dynamic expression patterns of these two enzymes during mouse heart development indicate that they may play an important role on heart development. However, there is a difference in spatiotemporal expression patterns between these two enzymes during heart development. The expression of p300 is earlier and more predominate, suggesting that p300 may play a more important role in embryonic heart development especially during cardiac precursor cell induction and interventricular septum formation.

## Background

The heart is the first functional organ during embryogenesis and its formation is initiated in a region of anterior mesoderm known as the cardiac crescent at about embryonic day 7.5 (E 7.5) in mice. Soon after their specification, the cardiac crescent from cardiac precursor cells converges along the ventral midline of the embryos to form the linear heart tube at around day E 8.5, which undergoes looping, chamber growth, specification and ultimately four-chamber formation [1,2].

Chromatin-remodeling and chromatin-modifying factors play a critical role in the process of cardiogenesis [3]. Histone acetyltransferases (HATs) and chromatin remodeling complexes facilitate chromatin opening and promote gene transcription [4]. The transcriptional co-activator p300 and CBP are HATs that regulate gene expression by acetylating histones and play central roles in a wide range of cellular processes during heart development [5-7]. CBP and p300 are highly homologous proteins that are well conserved among eukaryotic cells and have been considered to be functionally equivalent [8]. However, the reports from recent studies indicate that the two proteins may have different distribution patterns and functions in embryogenesis [9-14].

The role of CBP and p300 during the development was first revealed by the observations that human CBP gene was disrupted in a dominant genetic disorder, Rubinstein-Taybi Syndrome (RTS), which was characterized by craniofacial and limb defects, mental retardation as well as developmental anomalies of the eye, heart, kidney, lung, skin and testes [15]. In the subsequent gene disruption experiments in mice, it has been reported that mammalian development was tightly relevant to the CBP and p300 since knockout of p300 in mice resulted in an embryonic death of homozygous mutants during the days E 9 and E11.5 manifesting the defects in neurulation, cell proliferation, and heart development [16]. The CBP null animals exhibit a phenotype very similar to that observed in the p300 null mice. The homozygous mutants of CBP died on days E10.5-E12.5 with a massive hemorrhage in central nervous system, cranial neural tube closure defects and a developmental retardation in both primitive and definitive hematopoiesis. However, abnormal heart formation was not observed in CBP-deficient embryos [17]. Inactivation of acetyltransferase (AT) domain of murine p300 or CBP using a knock-in approach resulted in an increase of embryonic or neonatal lethality in mice. In addition, p300 AT domain mutations can cause multiple defects in the heart, lung and intestine formation, indicating that p300 AT plays a critical role in organogenesis [18]. p300 and CBP are generally present in mouse oocytes and pre-implanted embryos, and they experience the specific pattern of trafficking from the cytoplasm to the nucleus at different stages during the growth of oocytes [12].

Although it is well established that CBP and p300 are involved in transcription and development, scant information is available about the expression patterns and distributions of these two HATs in the hearts during the development. In the present study, we have systematically analyzed the p300 and CBP expressions during mouse cardiogenesis using RT-PCR and immunohistochemical techniques. The dynamic expression patterns of these two enzymes during mouse heart development indicate that they may play an important role on heart development. The expression of p300 is earlier and more predominate, suggesting that p300 may play a more important role in embryonic heart development especially during cardiac precursor cell induction and interventricular septum formation.

## Materials and methods

### Experimental animals

28–32 g KM mice were purchased from Experimental Animal Center of Chongqing Medical University. Animals were mated at 5:00 pm and females examined for a vaginal plug the following morning. The noon of the day when a vaginal plug was confirmed was considered as E0.5. Female mice were killed by cervical dislocation. The whole embryos at E7.5-E9.5 were collected. The whole hearts from embryonic mice at E10.5-E18 and from 1-day-old neonatal mice or from adult mice were collected accordingly. All procedures were approved by the Animal Care and Use Committee at the Chongqing Medical University (Chongqing, China)

### Immunohistochemical experiments

For immunohistochemical experiments, 3 embryos or 3 embryonic hearts or hearts from different mice at the same developmental stage were used and the embryo or heart samples were immediately fixed in fresh neutral formalin (37%–40% formalin, three-distilled water 880 ml, NaH_2_PO_4 _4 g, Na_2_HPO_4 _13 g) for 3–48 hours depending on the stage of the samples and embedded in paraffin wax. Thereafter, the specimens were sectioned at a thickness of 7 μm. Sections were deparaffinized, hydrated through gradual ethanol steps, and briefly rinsed in water. Endogenous peroxidases were inactivated by incubating the slides in 3% H_2_O_2 _(30% H_2_O_2 _10 ml, methanol 90 ml) for 10 minutes. Antigens retrieval was performed by boiling the slides in citrate buffer solution (trisodium citrate 3 g, citric acid 0.4 g, 1000 ml three-distilled water, PH 6.0) for 3 times and each time was separated by an interval duration 8 minutes. The unspecific protein binding sites on the sections were preblocked with 10% goat serum for 10 min. Slides were incubated with primary antibodies. CBP and p300 primary antibodies were purchased from Abcam Inc. (Cambridge, MA). Mouse monoclonal anti-p300 (ab3164) was diluted to 1:25 in PBS. Rabbit polyclonal anti-CBP (ab32646) was diluted to 1:700 in PBS. Samples were incubated with respective antibodies for overnight at 4°C and samples without primary antibodies were used as controls. Slides were then incubated with corresponding biotin-conjugated secondary antibody and HRP-conjugated third antibody for 30 min at 37°C in a wet box, respectively. Color reaction was generated for 3–5 min with DAB and stopped by rinsing the slides in distilled water. Finally, the slides were re-stained with hematoxylin and sealed with neutral gum. All slides were viewed using a Leica TCS SL microscope (Leica LCS Version 2.0).

### Total RNA isolation and RT-PCR analysis

Total RNA was isolated and reverse transcribed from pooled hearts at each stage (5–10 hearts per stage), ranging from E10.5 to E18 embryos, and from neonatal day 1 (n = 3) or from adult mice (n = 1) according to a reference previously described [19]. The extraction was performed using an Eukaryotic Perfect RNA Extract Kit (Genemega, China) following the manufacturer's protocols. Contaminating endogenous RNase was removed by treatment with DEPC (Amresco, USA) for 8 hour at room temperature. First-strand cDNA synthesis was carried out at 42°C for 1 hour using 6 μl of total RNA, oligo-dT primers and Superscript RNase H-reverse transcriptase (Promega Inc, Madison, WI.) according to the manufacturer's instruction. PCR was performed using 2 μl of First-Strand reaction mixture. As a negative control for genomic DNA contamination each sample was subjected to the same reaction without reverse transcriptase.

The specific primers were designed to detect mouse p300, CBP and β-actin as follows: p300 (forward) 5'-GCCAAGTATGCCAACCCTAA-3';

(reverse), 5'-TGTTCATTTGCTGAGCTTGG-3'.

CBP (forward) 5'-TGGAGTGAACCCCCAGTTAG-3';

(reverse) 5'-TTGCTTGCTCTCGTCTCTGA-3'.

β-actin (forward), 5'-TAGCCACGCTCGGTCAGGATCTTCAT-3';

(reverse), 5'-ACCAACTGGGACGACATGGAGAAGATC-3'.

Amplification conditions for p300 were a 7 minute hot start at 95°C followed by 35 cycles of 95°C for 1 minute, 60°C for 40 sec, and 72°C for 1 minute and a final extension at 72°C for 5 minute. The conditions for CBP were a 5 minutes hot start at 94°C followed by 35 cycles of 94°C for 1 minute, 54°C for 30 sec, and 72°C for 30 sec and a final extension at 72°C for 5 minutes. Conditions for β-actin were a 5 minutes hot start at 94°C followed by 30 cycles of 95°C for 1 minute, 58°C for 40 sec, and 72°C for 40 sec and a final extension at 72°C for 5 minute. All amplification products were resolved in a 1.5% agarose gel and sequencing confirmed the identity of each PCR amplified product and electrophoresis and the bands were analyzed with a Gelatin image formation meter (Bio-Rad) and Quantity One Version 4.4. The quantity of β-actin was used as an internal control to normalize the relative expression levels of p300 mRNA and CBP mRNA. Each experiment was repeated at least three times.

### Western Blotting

Nuclear proteins from fresh E10.5 embryo hearts were extracted using the Nuclear Extract Kit (ActiveMotif, Inc, Carlsbad, CA) according to manufacturer's instructions. Nuclear proteins were separated and electrophoresed on 6% Bis-Tris polyacrylamide gels and then transferred to a PDVF membrane. Blots were blocked in TBS plus 5% nonfat milk for 1 h. Then the blots were probed with either antibody against p300 (Abcam, ab3164, 1:200 dilution) or CBP (Abcam, ab32646, 1:1000 dilution) at 4°C overnight, and HRP conjugated goat anti-IgG antibody was used as the secondary antibody. Protein bands were revealed with an Enhanced Chemiluminescence Luminal reagent (Santa Cruz, CA), scanned and analyzed with Quantity One Version 4.4 software.

### Statistical analysis

Data were expressed as mean ± standard deviation (SD) and statistical differences in measured mRNA level between various experimental groups were assessed using an independent F-test with SAS (version 9). All tests used a α level of 0.05.

## Results

### Distributions of p300 and CBP during heart development

**Figure 1:**
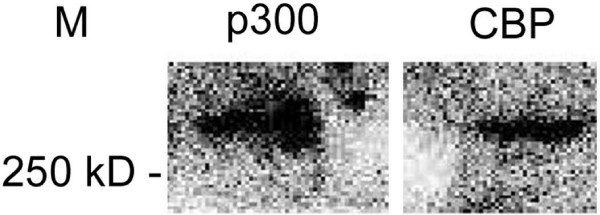
**CBP and p300 are detected by Western blotting in mouse embryonic heart samples**. Representative Western blots indicate that p300 and CBP were recognized by the specific antibodies against p300 and CBP respectively in nuclear protein extracts from E10.5 embryonic hearts. The experiments were performed as described in "Material and Methods" and the reaction was revealed by enhanced chemiluminescence assays (ECL).

The specificity of the antibodies was confirmed using nuclear protein extracts of E10.5 embryo hearts by Western blotting. As shown in Figure 1, both p300 (A, B) and CBP (C, D) protein expression was detected in E10.5 embryo hearts using the antibodies described in the "Materials and Methods".

We further analyzed the distribution of p300 and CBP proteins using immunohistochemical techniques in mouse heart tissues from the early embryonic stages (E7.5) to the adult stages.

**Figure 2:**
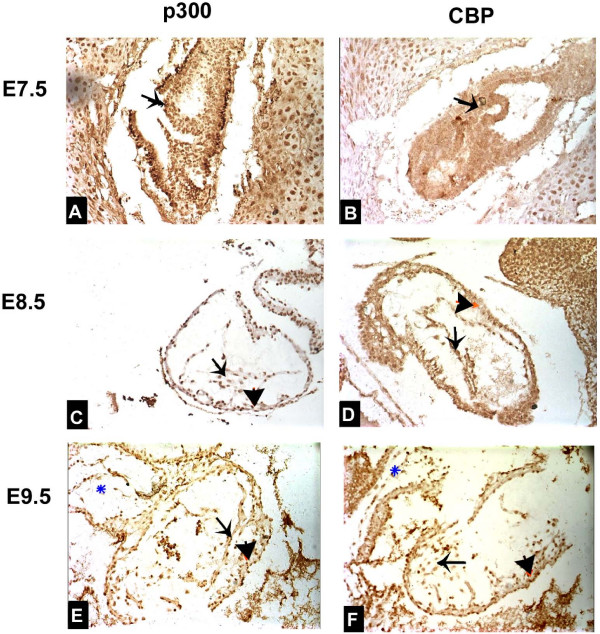
**Protein expression patterns of p300 and CBP in early embryonic hearts**. Lateral view of E7.5 embryo shows a high expression of p300 in cardiac crescents (arrow) at the anterior of the embryo (A). No expression of CBP was detected in cardiac crescents of a E7.5 embryo (arrow)(B). Lateral view of E8.5 embryo shows a high expression of p300 in linear heart tube at midline of the embryo including endocardium primordium (arrow) and myoepicardial mantle (arrowhead) which are the presumptive regions of myocardium and epicardium (C). Lateral view of E8.5 embryo shows a relatively low expression of CBP in the endocardium primordium (arrow) and myoepicardial mantle (arrowhead)(D). Lateral view of E9.5 embryo shows a high expression of p300 in looping heart tube at the left of the embryo including endocardium primordium (arrow) and myoepicardial mantle (arrowhead)(E). Lateral view of E9.5 embryo shows a relatively low expression of CBP in the looping heart tube including endocardium primordium (arrow) and myoepicardial mantle (arrowhead). Magnification: ×200.

We showed that during the early embryonic heart development stages (E7.5-E9.5), p300 was highly expressed in crescent-shaped cardiogenic plate (future myocardium) at E7.5 (Figure 2A), while no CBP expression was detected in this region at the same stage (Figure 2D). We also showed that p300 was highly expressed at E8.5 in the fusing heart tubes including endocardium primordium and myoepicardial mantle, the presumptive regions of myocardium and epicardium (Figure 2B). Similarly, we found high level expression of p300 at E9.5 in the looping heart tubes which include all cardiac compartments, i.e. primary atrial and ventricular myocardium as well as outflow tract (Figure 2C). CBP was also observed in these regions at E8.5 and E9.5, however, the protein level was much less compared to p300 (Figures 2E & 2F).

**Figure 3:**
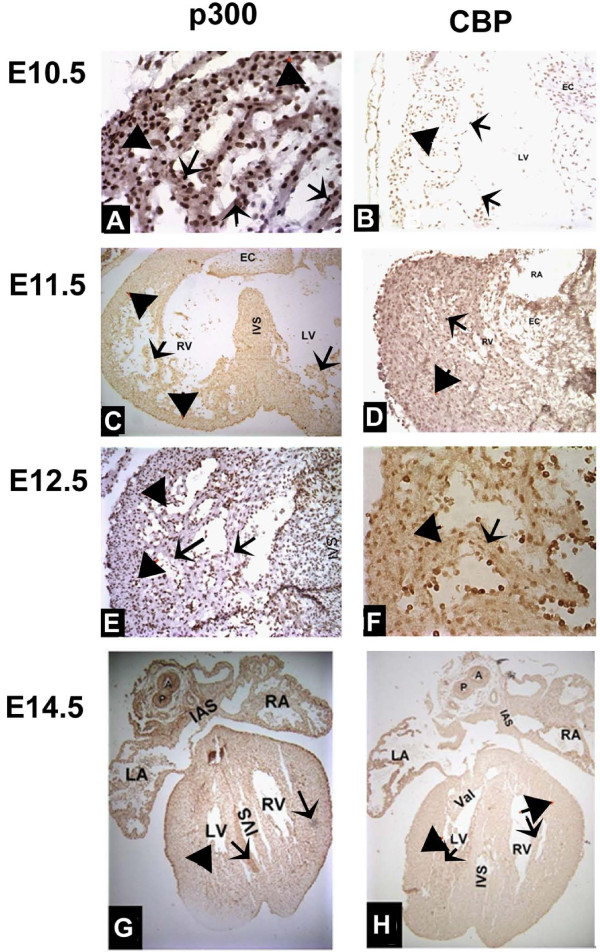
**Expressions of p300 and CBP in embryonic hearts during chamber formation and septation stages from E10.5 to E15.5**. E10.5 embryo heart shows a high p300 expression (A) and a relative low expression of CBP (B) in most cardiac compartments, in particular in the trabeculae (arrow) and ventricular myocardium (arrowhead). C shows a high expression of p300 at E11.5 in atria and ventricle walls (arrowhead), ventricular trabeculae (arrow). D shows that a high expression of CBP was found at E11.5 in atrioventricular myocardium (arrowhead), a relatively low expression of CBP was found in trabeculae (arrow) and OFT. At E12.5, p300 is highly expressed in atria and ventricle walls (arrowhead), ventricular trabeculae (arrow)(E). A similar CBP expression pattern is shown in E12.5 embryos (F) in cardiac walls (arrowhead) and trabeculae (arrow). In E14.5 embryonic heart, p300 is highly and extensively expressed in walls, ventricular trabeculae, muscular IVS and nascent membranous IVS, nascent pulmonary artery and aorta (G). At the same stage, CBP is highly expressed in atrioventricular myocardium and lowly expressed in atrioventricular valves, trabeculae and OFT (H).

During the chamber specific forming and septation stages(E10.5-E15.5), the mouse heart morphological changes were observed as follows. The mouse heart was unseptated at E9.5, and looped tube formed which had three oncoideses, i.e. bulbus cordis, primary ventricle and primary atria from top to end. As heart developed further, confined atrioventricular canal (AVC) formed between atria and ventricle. Meanwhile, the endocardial cushions (EC) became visible at E10.5 in the AVC. At this stage, ventricular trabeculae were evidently visible within the presumptive right ventricle (RV) and left ventricle (LV). By E11.5, there had been evidently visible EC and gradually forming primary atrial septum (PAS) as well as considerable growth of the muscular interventricular septum (IVS) so that the RV and LV were now separated apically, which, at this stage, the ridges within the outflow tract (OFT) had started to appear and face to fuse, and the mesenchymal swellings that would become the atrioventricular valves were visible. By E13.5, second atrial septum had completely formed, and aortico-pulmonary septum had formed so that the pulmonary artery and ascending aorta were separated. Meanwhile, atrial trabeculae were visible. By E14.5, membranous IVS resourced from EC had fused to muscular IVS. By E15.5, tricuspid and mitral valves had formed at the right and left atrioventricular foramen, respectively. As shown in Figure 3, in these stages, p300 was highly expressed in developing chamber walls, i.e. ventricular myocardium, atrial myocardium and interatria septum (IAS), IVS, trabeculae, atrioventricular EC and OFT which gradually developed into pulmonary artery and ascending artery (Figures 3A–I). However, CBP distribution was not so even as we found a higher distribution in atrioventricular myocardium and a relatively lower distribution in atrioventricular valves, trabeculae and OFT and a very low distribution in the developing muscular IVS and membranous IVS (Figures 3J–R).

**Figure 4:**
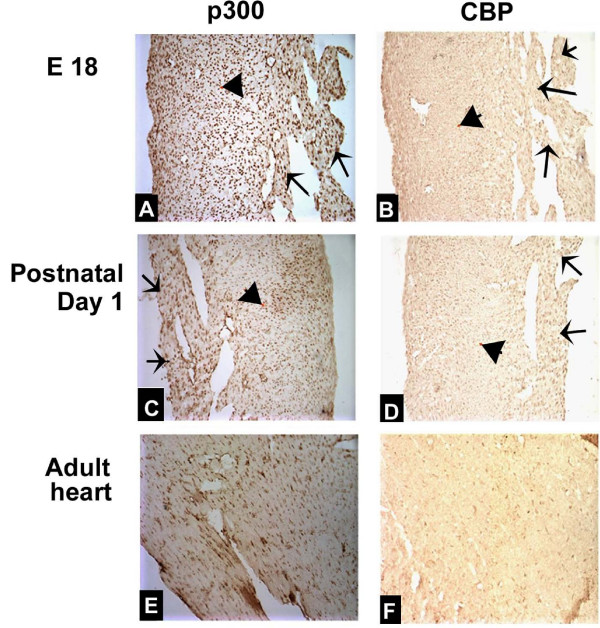
**p300 and CBP expressions in mouse hearts at late fetal stages, postnatal day 1 and adulthood stages**. p300 is highly expressed in myocardium (arrowhead) and trabeculae (arrow) but relatively lowly expressed in septum and valves in E18 embryonic heart (A) and in postnatal day 1 heart (C). A relatively low expression of CBP in myocardium (arrowhead) and trabeculae (arrow) was shown in E18 embryonic heart (B) and postnatal day 1 heart (D). In adult mouse hearts, both p300 (E) and CBP (F) expression were low.

At late fetal developmental stages (from E16.5 till born), the heart has complete four chambers separated by IAS, IVS and the atrioventricular valves. As the heart developed, the myocardial cell number increases, which resulted in a bigger heart. In embryonic hearts (E16.5 and E18), we found that p300 was highly expressed in myocardium and trabeculae, but relatively lowly expressed in septum and valves (Figures 4A & 4B). We also showed that CBP expressions were relatively low in myocardium and trabeculae, as well as in muscular IVS and lower expressions in membranous IVS (Figures 4G & 4F). Similar findings were observed in the postnatal day 1 hearts (Figures 4C, D, H, I).

In adult mouse hearts, relatively high expressions of p300 were observed in chamber walls (Figure 4E) and low expressions of p300 were found in septum and valves (data not shown). However, low expressions of CBP were observed in chamber walls (Figure 4J) and muscular IVS. CBP in membranous IVS was extremely low (data not shown).

### p300 and CBP mRNA expressions during mouse heart development

**Figure 5:**
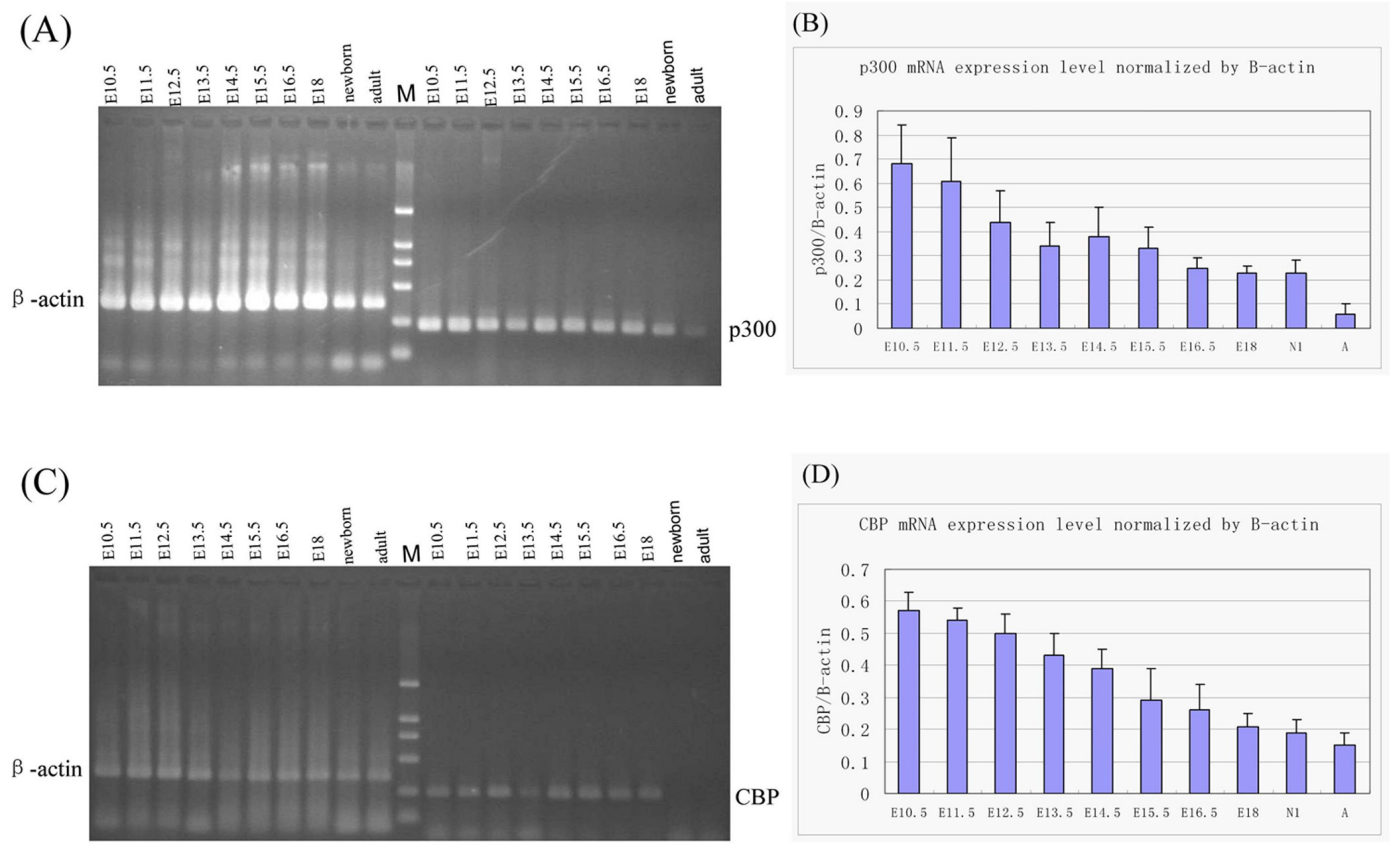
**p300 and CBP mRNA expression levels during mouse heart development**. RT-PCR data show that p300 (A) and CBP (C) mRNA expressions can be detected at various embryonic stages as well as in postnatal and adult stages. M represents DNA marker. (B, D) The line graphs summarize p300 (B) and CBP (D) relative expression levels corresponding to panel A and C, respectively. Data are expressed as a ratio of p300 or CBP mRNA to β-actin mRNA concentrations. Results are averaged from three independent experiments in each stage.

We further investigated p300 and CBP mRNA expression levels during mouse cardiogenesis using RT-PCR techniques. This enabled a comparison of the semi-quantitative expression of p300 and CBP mRNA level at different developmental stages to be made. These data were expressed as a ratio of p300 or CBP to β-actin expression levels. The earliest time of collecting cardiac tissues for RT-PCR was day E10.5 which was the earliest stage to gain the completely isolated heart. The expression levels of both p300 and CBP gradually diminished after day E12.5 but a weak expression was still observed on day 1 after birth and in adult hearts (Figure 5). The mRNA expression data are consistent with the results we obtained from the immunohistochemical studies.

## Discussion

The current model of mammalian heart development indicates that the crescent primary heart primordium derived first from cardiac precursor cells (on day E7.5 in mice) undergoes fusion to form primary linear heart tube (on day E8.5). Then the tube loops to form primary chamber (E9.5). The chamber separation occurs on E10.5 – E15.5 to ultimately form a four-chambered heart and the heart continue to grow till the consummation. During the process, cardiac cell differentiation plays a critical role to allow certain genes to be expressed at a given stage of the development [20]. The selection applies to either the activation or the repression of certain cardiac genes. Modification of histone acetylation by HATs is an important pattern to control the gene expressions. Both p300 and CBP are the members of the HATs family and they are a pair of closely homologous co-activators that are often referred to as a single entity [21]. Although it has been reported that p300 and CBP are essential for multi-organogenesis [8-14], little is known about their locations and dynamic expression in the heart during the development. In this study, we have investigated the distributions of these two enzymes in the heart during the heart formation from the very beginning of the cardiac crescent forming at E7.5 to adult heart using immunohistochemistry and RT-PCR to better understand the roles of these two enzymes on cardiogenesis. Our RT-PCR data have shown that both CBP and p300 are present in the heart during the development from E10.5 till adulthood with a similar dynamic variance and a high expression at the early stages of chamber septation. In addition, we have observed that p300 is highly expressed in the heart from E10.5 to E11.5 with a peak expression at E10.5 whereas CBP is highly expressed from E10.5 to E12.5 and with a peak expression at the same time. Expression of both p300 and CBP is gradually decreased in the subsequent heart development stages till the postnatal and adulthood stages, suggesting that p300 and CBP are required for gene expression during the heart development. Furthermore the findings also support the notion that both enzymes have a concomitant transcriptional regulation during cardiogenesis.

However the immunohistochemical data reveal different distribution patterns of p300 and CBP in early stages of heart development, suggesting a potential functional difference between these two enzymes. In the stage of cardiac precursor cells induction (day E7.5), p300 has a much higher distribution than CBP in crescent-shaped cardiogenic plate, indicating that p300 probably plays a more important role on cardiac progenitor cells induction and migration. p300 is highly distributed in all areas of heart including cardiac walls, septum, trabeculae, developing cardiac cushions and valves during the subsequent stages from the fetal development till the postnatal day 1. In adult mouse hearts, a relatively high presentation of p300 is observed in the walls and a lower distribution is seen in the septum and the valves. Unlike p300, CBP has a different distribution pattern. A relatively high presentation of CBP is found in the early linear cardiac tube at E8.5 and in the looping cardiac tube at E9.5. From the chamber septation and late heart development stages till the postnatal day 1. CBP is found with a high concentration in atrioventricular walls and with a less content in atrioventricular valves, trabeculae, and a very low level in developing muscular IVS and a lower expression in forming membranous IVS. In adult mouse hearts, a low level of CBP is observed in walls and muscular IVS and extremely low content of CBP is observed in membranous IVS. These findings suggest that both p300 and CBP may participate in the morphogenesis of heart, which is in consistent with the results from the RT-PCR experiments in this study. However p300 may have more important functions than CBP for heart development, since it has a broader distribution in the heart especially during the stages of trabeculation and cardiac septum formation. These observations are consistent with the previous studies [22,23].

In general, the expression of p300 and CBP is ubiquitous in mammalians [9,16]. The phenotypes observed in transgenic mice harboring mutated CBP or p300 indicate that both CBP and p300 are essential for normal embryogenesis [16]. Our experimental results suggest that p300 is ubiquitously expressed in developing hearts and throughout postnatal as well as in adult hearts. The distribution pattern of p300 in the heart during development is similar to that of CBP with an expectation that no CBP is found on day E7.5.

Although p300 and CBP have great similarity in both biochemical structures and functions, increasing researches have shown that CBP and p300 might possess distinct developmental functions due to differences in their distribution and expression patterns. Animals with deficiency of p300 or CBP exhibited abnormal phenotypes suggesting that CBP and p300 cannot be replaced in the homozygous mutant mice [16,17]. It is known that patients suffering from Rubinstein-Taybi syndrome, an autosomal dominant syndrome characterized by abnormal growth and mental retardation as well as abnormal heart development, commonly have CBP gene mutations (resulting in lower CBP levels) while the p300 gene of these patients is considered to be normal [15,24,25]. In F9 cells, retinoic acid induced differentiation is dependent on p300 but not on CBP [26]. CBP is found to be localized mainly within the oocyte nucleus while p300 is localized in the cytoplasm after two-cell stage and they could not replace each other in these processes [12]. Moreover, a full complement of CBP, but not p300, is required for normal hematopoietic differentiation [27,28]. In consistent with all these reports, our results indicate that there are differences between p300 and CBP in both distribution and temporal expression pattern during the heart development.

## Conclusion

Our data show that the expression of CBP and p300 is developmentally regulated during mouse cardiogenesis. There are differences between CBP and p300 in both tissue distributions and mRNA expression levels during heart development. These observations shed new light on the function of CBP and p300 during heart development and provide us with a molecular basis for further understanding the mechanisms underlying cardiogenesis and some congenital heart diseases.

## Competing interests

The authors declare that they have no competing interests.

## Authors' contributions

GC carried out the immunohistochemical studies and drafted the manuscript. JZ carried out the histological studies for observation of the heart developmental morphology. TL carried out western blotting assays and participated in the sequence alignment experiments. GW carried out the molecular genetic studies using RT-PCR and participated in the sequence alignment experiments. HS performed statistical analysis and participated in drafting the manuscript. XH edited and revised the manuscript and participated in the design of the study. JT organized the study and participated in study design and coordination. All authors read and approved the final manuscript.
